# Relationship Between Age and Grip Strength, Phase Angle, and Impedance Rate Measured by Bioelectrical Impedance Analysis in Japanese Individuals Aged 15–18 Years

**DOI:** 10.33549/physiolres.935774

**Published:** 2026-06-01

**Authors:** Kazushige OSHITA, Yujiro ISHIHARA, Takeshi HORAI, Tadashi UNO, Satoki MURAI

**Affiliations:** 1 Department of Human Information Engineering, Okayama Prefectural University, Japan; 2 Center for Fundamental Education, Okayama University of Science, Japan; 3 Department of General Education, National Institute of Technology (KOSEN) Suzuka College, Japan; 4 Center of Liberal Arts and Science, Sanyo-Onoda City University, Japan

**Keywords:** Muscle quality, Muscle mass, Muscle strength, Puberty, Adolescence

## Abstract

Although muscle mass increases during puberty, it plateaus in girls and slowly increases in boys after around 15 years of age. This study investigated the relationship between phase angle and impedance ratio as indicators of muscle quality and age in Japanese individuals aged 15–18 years and examined their relationship with grip strength. Participants included 838 high school, college, or university students (534 boys, 304 girls). Body composition, phase angle, and impedance ratio were measured using a multi-frequency bioelectrical impedance analyzer. Phase angle was calculated from the resistance and reactance to a 50-kHz alternating current, while impedance ratio was calculated from the impedance to 5- and 250-kHz currents. Grip strength was measured using a Smedley dynamometer. Significant effects of age were observed for body size, fat-free mass, grip strength, phase angle, and impedance ratio in the boys. Compared to 16-year-olds, 17-year-olds showed a significantly higher phase angle and significantly lower impedance ratio in whole body, upper limb, and lower limb. In contrast, no significant main effects of age were observed for any of these measures in the girls. Phase angle and impedance ratio were significantly correlated with grip strength at each age in both sexes. These results suggest that phase angle and impedance ratio can be used to assess muscle strength at any age during late adolescence. However, age-specific values may be preferable for boys in late adolescence when using the reference or percentile values for phase angle and impedance ratio.

## Introduction

During puberty, dramatic hormonal fluctuations and rapid increases in body size (the most widely used anthropometric measurements are body height and body mass [1]) are accompanied by increases in the main body components (total body fat, lean body mass, and bone mineral content) [2]; however, considerable sexual dimorphism exists [2,3]. During early to mid-puberty, boys and girls experience increases in both fat mass (FM) and fat-free mass (FFM) [3]. FFM increases rapidly in boys, particularly between the ages of 12 and 15 years, but steadily overall between the ages of 8 and 18 years [2]. Although FFM in girls also increases during puberty, the maximum FFM is attained by 15 or 16 years of age [4,5] and remains relatively unchanged thereafter [2,3,6]. Therefore, at age 15 years and beyond, sex-based differences in the absolute and relative amounts of FM and FFM become even more pronounced [7]. Furthermore, such changes in body composition during puberty are markers of the metabolic changes that occur during this period of growth and maturation and hold key information regarding an individual’s current and future health [2].

The muscle tissue comprises both intracellular and extracellular components. Consequently, recent studies have focused not only on muscle mass, but also muscle cellular components. Using the bioelectrical impedance analysis (BIA) method, which estimates body composition using the impedance of a weak alternating current (AC) flowing through the body [8], phase angle (PhA) and impedance ratio (IR) are also calculated from raw bioelectrical impedance data [9–11]. Impedance is a complex number that comprises the resistance (R) or purely resistive component (water and electrolytes in fluids and tissues) and the reactance (Xc) or capacitative component in tissues (cells and tissue interfaces) [9]. The ESPEN – European Society for Clinical Nutrition and Metablism “Blue” Book introduces them as follows [10]; The PhA, which is calculated from the R and Xc, is a reasonably good indicator of body cell mass, but it also indicates cell membrane integrity and function. IR is another method of evaluating cell membrane function, which uses a multifrequency BIA device. Resistance at low-frequency AC (<50 kHz) is evaluated to determine extracellular water, while resistance at high-frequency AC (>100 kHz), where cell membranes are penetrated, is evaluated to determine total body water. IR is calculated as the ratio of high-frequency to low-frequency AC impedance. Under normal conditions, the ratio of impedance at 200 kHz to that at 5 kHz is always less than 1; however, when there is an alteration or impairment to the function of the cell membrane, this ratio approaches unity.

The characteristics of these bioelectrical impedance parameters suggest that PhA is responsive to changes in the body’s internal environment [12]. Therefore, PhA has strong potential as a practical biomarker for assessing nutritional status, guiding physical training and monitoring early signs of metabolic imbalance [12]. In the field of exercise training, PhA has recently gained attention in sports science, in addition to being used to assess body composition [13]. A recent study reported that PhA can be used to assess differences in competition and activity levels that are not represented by body size or muscle mass [14]. Further, a previous study reported that PhA has a stronger association with and is a marker of muscle performance in youth [15]. A previous review of young athletes also reported that researchers and practitioners could use PhA to monitor their general health status, muscle performance, and muscle quality [16]. Regarding the assessment of nutritional status, a recent review reported that, because most studies have observed an association between PhA and objective indicators of nutritional status in children, PhA is an easily obtainable parameter that can be used to diagnose malnutrition [17]. Since such various applications have been proposed, PhA reference or percentile values for younger generations have been reported in large-scale studies and systematic reviews [18–21].

However, normal PhA values vary by ethnicity [19,21], with lower values observed in Asians [21]. Therefore, age-specific percentile values of PhA and IR for Japanese individuals have been reported, although those aged <20 years were grouped together as 15–19-year-olds [22]. From the perspective of muscle growth and development, boys experience sharp growth in FFM until around 15 years of age, followed by a gradual increase until around 18 years of age [2,4]. In contrast, muscle mass in girls remains relatively stable after approximately 15 years of age [2,3,6]. Therefore, PhA and IR may exhibit age-related differences among 15–18-year-old boys while remaining stable in girls. If age-related differences are observed, PhA and IR should be exhibited age-specific percentile values for the 15–18-year-old age group. This study aimed to investigate the relationship between body composition, raw bioelectrical impedance indices (PhA and IR), and muscle strength with age among Japanese students aged 15–18 years and provide percentile values for PhA and IR.

## Methods

### Participants

**Fig. 1:**
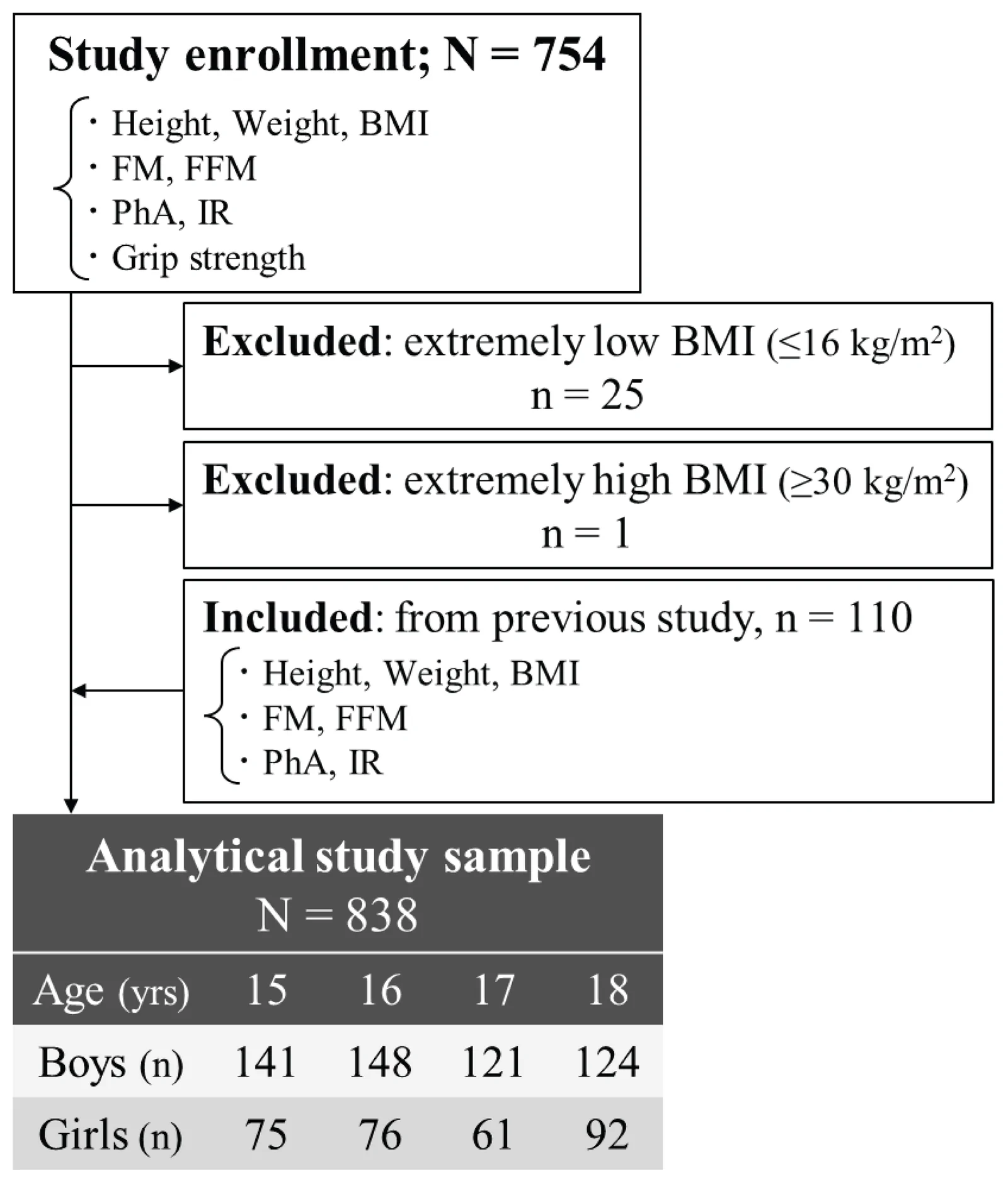
Flow diagram outlining participant selection for this study. BMI, body mass index; FFM, fat-free mass; FM, fat mass; IR, impedance ratio; PhA, phase angle.

This study included data from 838 high school, college, and university students in Japan aged 15–18 years (534 boys, 304 girls) (Fig. 1). First, data from 110 individuals were collected from our previous study [22]. This study also included the following additional data. To avoid seasonal measurement variations, body size, body composition, and grip strength were measured in 754 students between April and May 2025 for alignment with the survey period (April to June) of the School Health Examination Survey of Japan [23]. Data from 728 individuals (487 boys, 241 girls) were included after the exclusion of those with an extremely low (≤16.0 kg/m^2^) or high (≥30.0 kg/m^2^) body mass index (BMI). This BMI range was derived using Z-score tables of WHO-BMI for age standards for children and adolescents between 5 and 19 years; for the 15–18 year age range (180–227 months), median −2 standard deviation (SD) corresponds to 16.0–17.5 kg/m^2^ for boys and 15.9–16.5 kg/m^2^ for girls, while median +2SD corresponds to 27.0–29.7 kg/m^2^ for boys and 28.2–29.7 kg/m^2^ for girls. This study was reviewed and approved by the Research Ethics Committee of Okayama University of Science (approval number: 7–7) and informed consent was obtained from all the participants and their parents prior to the examination.

### Body size and body composition

Body height was measured to the nearest 0.1 cm using a stadiometer. Body mass was measured to the nearest 0.1 kg using a BIA measuring device. BMI was calculated by dividing body mass in kilograms by height in meters squared (kg/m^2^).

Body composition (FM and FFM), PhA, and IR were measured using a standing 8-electrode multi-frequency BIA (MC-780A-N; TANITA, Tokyo, Japan). The device measures R and Xc by applying AC of <90 μA at frequencies of 5 kHz, 50 kHz, and 250 kHz from electrodes on the soles of the feet and the palms of the hands. The participants wiped the palms of their hands and soles of their feet with alcohol-free wet wipes to clean and moisten the electrode contact areas. Next, they stepped onto the electrode portion of the machine and grasped the electrodes with both palms to enable the measurements. The participants were asked to urinate and defecate before the measurements and avoid eating immediately before the assessments.

The BIA analyzer recorded Xc, R, and PhA along with body composition. PhA was calculated using Xc and R at 50 kHz AC [24] as the arctangent between the R and Xc, =(Xc/R)×(180°/π). Impedance was calculated from R and Xc, while IR was calculated from impedances for the 5- and 250-kHz ACs. PhA and IR were assessed as follows: for the whole-body, using measurements from the left body half; and for the upper and lower limbs, using the mean values from both limbs.

### Grip strength

Grip strength was measured using a Smedley grip dynamometer (TKK5001, Takei scientific instruments Co. ltd. Japan or 100 EKJ094, EVERNEW Inc., Japan) in accordance with the procedures of a survey of physical fitness and motor ability in Japan [25]. This was performed during the same period as the body size and composition measurements. The participants held the dynamometer with the display facing outward and adjusted their grip width such that the second knuckle of the index finger formed an approximate right angle. Each gripped the dynamometer with maximum force while standing upright and ensured that it did not touch their body or clothing. The measurements were performed twice for each hand, alternating between the right and left, and the highest value for each was recorded.

### Statistical analyses

The measurements were analyzed separately for the boys and girls. Each measurement value is expressed as mean ± standard deviation.

Age-related differences in each measurement value were examined using one-way ANOVA. When a significant main effect of age was observed, multiple comparisons were performed of 15- and 16-year-olds, 16- and 17-year-olds, and 17- and 18-year-olds using Bonferroni method. Effect sizes are expressed as d-values and were graded as follows: *d*<0.20, trivial effect; *d*=0.20–0.49, small effect; *d*=0.50–0.79, moderate effect; and *d*≥0.80, large effect. The relationship between PhA or IR and other variables was assessed using Pearson’s correlation coefficient (r). Statistical analyses were performed using StatFlex software (ver. 7.0.10; Artec, Osaka, Japan). Statistical significance was defined as *P*<0.05.

## Results

Table 1 presents the mean values of body size, BMI, FM, FFM, and grip strength by age. Table 2 presents the mean PhA and IR values for the whole-body, upper limbs and lower limbs by age. For boys, significant main effects of age were observed for all these measurement variables. Multiple comparisons revealed no significant age-related differences in height, BMI or FM. Body mass, FFM, grip strength, and whole-body PhA, were significantly higher, and whole-body IR were significantly lower, at 17 vs. 16 years of age (*P*<0.05). The effect sizes for body mass and FFM in boys aged 16–17 years were small, whereas those for grip strength and whole-body PhA, and IR were close to moderate (*d*=0.40–0.45). Further, significant differences in PhA and IR were also observed between 16- and 17-year-olds in the upper and lower limbs, though the effect size was moderate for the upper limbs (*d*=0.55–0.65) and small for the lower limbs (*d*=0.34). For the girls, no significant effect of age was observed on any of the measured variables. For all measurement variables, the effect sizes between the 15- and 16-year-olds, 16- and 17-year-olds, and 17- and 18-year-olds were small (*d*<0.40). These results indicate that, while no age-related differences were observed in body size, body composition, or muscle strength in girls, boys exhibited higher values at 17 vs. 16 years of age. Further, boys exhibited significantly higher PhA and lower IR in whole-body, upper limbs and lower limbs, at 17 years of age compared to 16 years of age.

Table 3 show the relationship between whole-body PhA or IR and other variables. PhA was significantly positively correlated with body mass, BMI, FM and FFM, while IR was significantly negatively correlated with these variables in boys (*P*<0.01). Height had no significant association with either PhA or IR. PhA was significantly positively correlated with body mass, BMI and FFM, while IR was significantly negatively correlated with these variables in girls (*P*<0.01). Height and FM had no significant association with either PhA or IR.

**Fig. 2:**
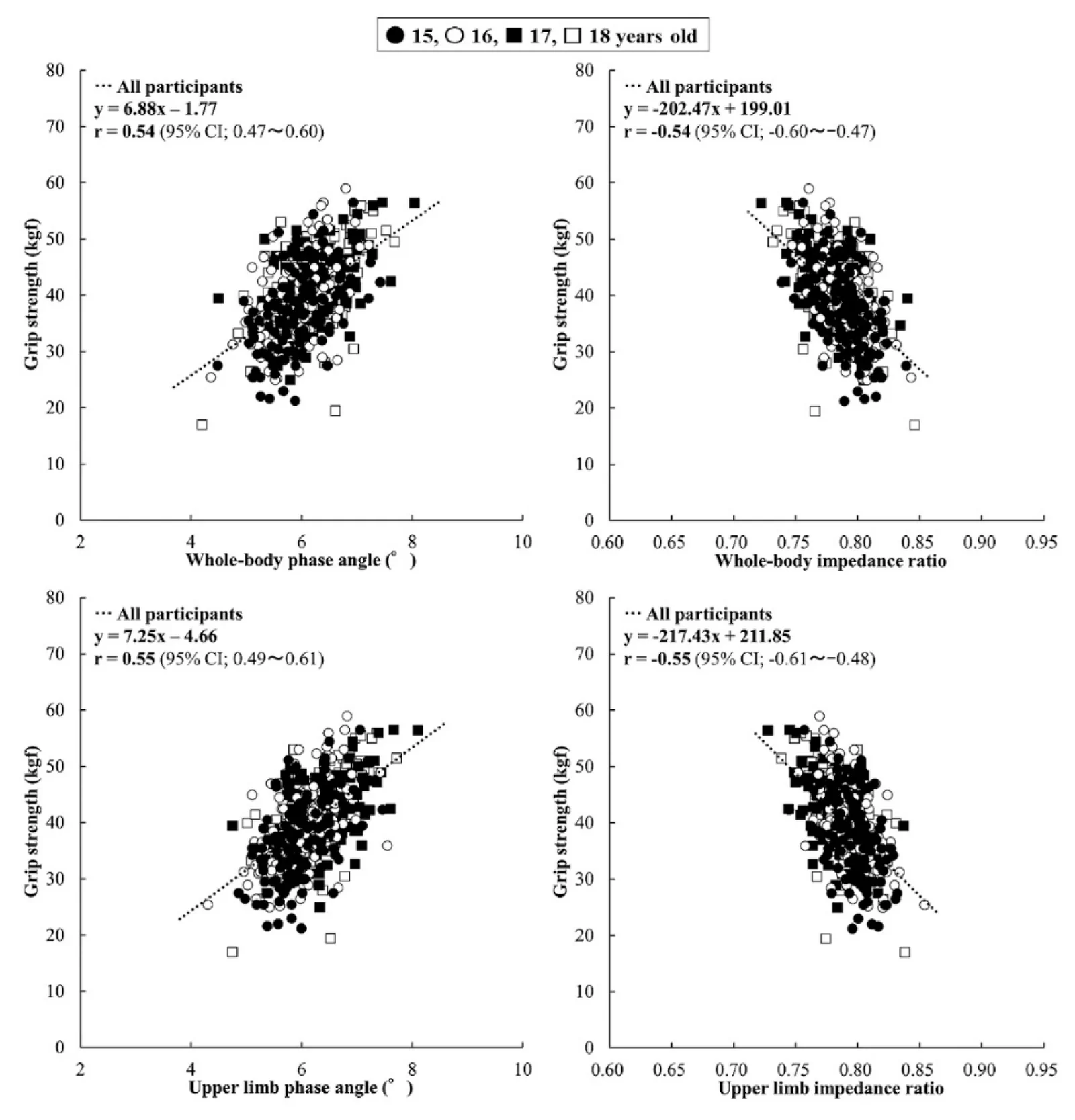
Relationship between grip strength and phase angle or impedance ratio in boys. Age-specific (ages 15, 16, 17, and 18) correlation coefficients (r): Whole-body phase angle; 0.51, 0.50, 0.48, and 0.54, Whole-body impedance ratio; −0.51, −0.52, −0.46, and −0.53, Upper limb phase angle; 0.49, 0.54, 0.46, and 0.57, Upper limb impedance ratio; −0.49, −0.56, −0.45, and −0.56, *P*<0.05 respectively.

**Fig. 3:**
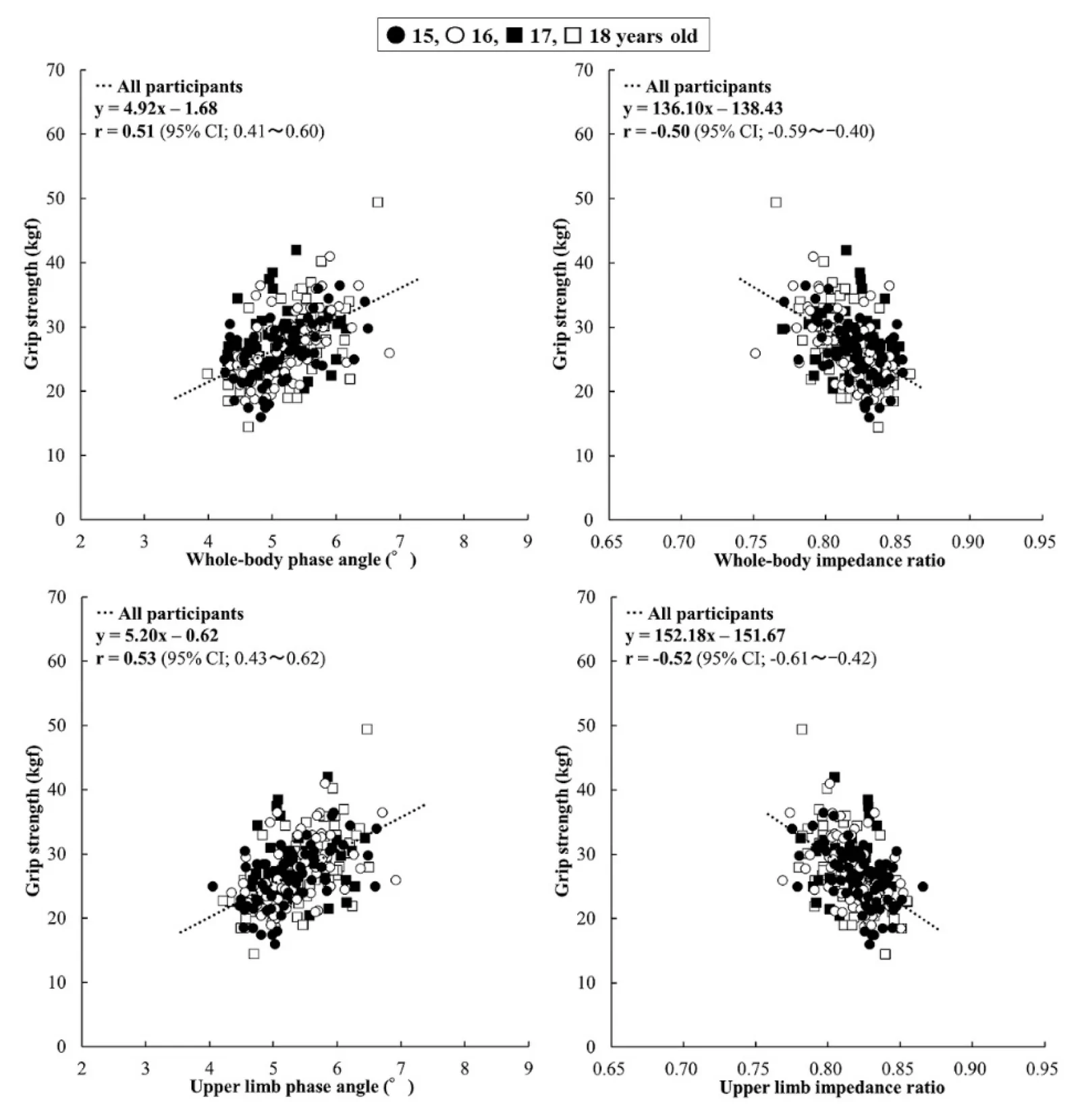
Relationship between grip strength and phase angle or impedance ratio in girls. Age-specific (ages 15, 16, 17, and 18) correlation coefficients (r): Whole-body phase angle; 0.53, 0.52, 0.41, and 0.54, Whole-body impedance ratio; −0.52, −0.47, −0.41, and −0.56, Upper limb phase angle; 0.57, 0.48, 0.47, and 0.57, Upper limb impedance ratio; −0.53, −0.46, −0.50, and −0.56, *P*<0.05 respectively.

Table 3 (bottom part), Figures 2 and 3 show the relationship between grip strength and PhA or IR, with closed circles, open circles, closed squares, and open squares representing 15-, 16-, 17-, and 18-year-olds, respectively. Across all boys (Fig. 2), grip strength showed a significant positive correlation with whole-body PhA (r=0.54, *P*<0.01) and significant negative correlation with whole-body IR (r=−0.54, *P*<0.01). At all ages, grip strength showed a significant positive correlation with whole-body PhA (r; 0.51 at age 15, 0.50 at age 16, 0.48 at age 17, 0.54 at age 18, *P*<0.01) and significant negative correlation with whole-body IR (r; −0.51 at age 15, −0.52 at age 16, −0.46 at age 17, −0.53 at age 18, *P*<0.01). Further, grip strength showed a significant positive correlation with upper limb PhA (r=0.55, *P*<0.01) and significant negative correlation with upper limb IR (r=−0.55, *P*<0.01). At all ages, grip strength showed a significant positive correlation with upper limb PhA (r; 0.49 at age 15, 0.54 at age 16, 0.46 at age 17, 0.57 at age 18, *P*<0.01) and significant negative correlation with upper limb IR (r; −0.49 at age 15, −0.56 at age 16, −0.45 at age 17, −0.56 at age 18, *P*<0.01). Across all girls (Fig. 3), grip strength showed a significant positive correlation with whole-body PhA (r=0.51, *P*<0.01) and a significant negative correlation with whole-body IR (r=−0.50, *P*<0.01). Further, at all ages, grip strength showed a significant positive correlation with whole-body PhA (r; 0.53 at age 15, 0.52 at age 16, 0.41 at age 17, 0.54 at age 18, *P*<0.01) and significant negative correlation with whole-body IR (r; −0.52 at age 15, −0.47 at age 16, −0.41 at age 17, −0.56 at age 18, *P*<0.01). Further, grip strength showed a significant positive correlation with upper limb PhA (r=0.53, *P*<0.01) and significant negative correlation with upper limb IR (r=−0.52, *P*<0.01). At all ages, grip strength showed a significant positive correlation with upper limb PhA (r; 0.57 at age 15, 0.48 at age 16, 0.47 at age 17, 0.57 at age 18, *P*<0.01) and significant negative correlation with upper limb IR (r; −0.53 at age 15, −0.46 at age 16, −0.50 at age 17, −0.56 at age 18, *P*<0.01). These results indicate that grip strength is related to PhA and IR regardless of age.

## Discussion

The mean body height and mass of girls in Japan’s latest School Health Examination Survey [23] were 157 cm and 51.1 kg, 158 cm and 52.0 kg, and 158 cm and 52.5 kg at ages 15, 16, and 17 years, respectively. Grip strength, as measured in the Survey on Physical Fitness and Motor Abilities of Japan [25], was 25.3 kgf, 26.0 kgf, and 26.6 kgf at ages 15, 16, and 17 years, respectively. These values are almost equivalent to those of the participants in this study, indicating that the girls in this study were representative of the general population of Japanese girls in terms of body size and muscle strength. FFM typically plateaus in girls at age 15 years or later [2,3,6]. No significant effects of age were observed for body size variables or FFM in this study. Moreover, no significant effect of age on grip strength was observed. Similarly, no significant effects of age were observed for PhA or IR in whole-body, upper limbs and lower limbs. Therefore, these findings suggest that significant age-related differences are not observed in girls aged 15–18 years in either FFM or muscle cellular state.

The mean body height and mass of boys in the School Health Examination Survey of Japan [23] were 169 cm and 59.0 kg, 170 cm and 60.5 kg, and 171 cm and 62.2 kg at ages 15, 16, and 17 years, respectively. Furthermore, the grip strength results from the Survey on Physical Fitness and Motor Abilities of Japan [25] were 37.0 kgf, 38.9 kgf, and 40.7 kgf at ages 15, 16, and 17 years, respectively. Although the participants in this study had slightly lower body mass, their heights and grip strengths were nearly equivalent to those in these surveys. Unlike girls, boys experience a sharp growth in FFM until approximately age 15, followed by a gradual increase until approximately age 18 [2,4]. In this study, significant effects of age on body mass and FFM were also observed in boys, with values being significantly higher at 17 years of age than at 16. The effect sizes in body mass and FFM between 16- and 17-year-olds were approximately 0.3. In contrast, whole-body PhA and IR were significantly higher at 17 than 16 years, with effect sizes close to moderate (*d*=0.44–0.45). Analyzed by body part, the effect size was moderate (*d*=0.55–0.65) for the upper limbs and small (*d*=0.34) for the lower limbs. These results suggest that the degree of difference between 16- and 17-year-olds in PhA and IR is greater than that in FFM. Therefore, the reference or percentile values for PhA and IR in boys should be presented separately according to age (as shown in the Table S1). In particular, values for 15–16- and 17–18-year-olds should be presented separately.

Previous studies reported an increase in PhA until the end of puberty in boys vs. a plateau in girls [26], findings that are consistent with the present results. This was explained by a previous study indicating that an increase in PhA in children and adolescents is associated with growth, probably because of an increase in body cell mass (BCM) with age [18]. Therefore, although FFM and PhA increased with age in boys in the present study, no age-related differences were observed in girls. IR reflects the ratio of intra- to extracellular components, and the elderly show higher values than adults [22]. As boys mature, they tend to have a greater number of intracellular components, which reflects an increase in BCM [27]. Therefore, although IR had also significant effects of age in boys in this study, no age-related differences were observed in girls.

Furthermore, grip strength was positively and negatively associated with PhA and IR, respectively. Similar results were found in adolescents, in whom higher physical fitness values were directly associated with a higher PhA [28]. Furthermore, a relationship between PhA and physical fitness was reported in Japanese adolescent athletes [29]. Muscle strength is directly related to muscle mass, which has high concentrations of body water, particularly the intracellular component, which partly explains the association between PhA or IR and muscle strength [28]. Therefore, the findings of this study suggest that PhA and IR are reflective indicators of muscle strength in both sexes at 15–18 years. However, while it is true that BCM is predominantly influenced by the cell content of larger organs, such as muscle tissue (myocytes account for up to 80 % of the BCM), the characteristics of other cells (e.g., adipocytes) may also affect the bioelectrical impedance variables [30]. Therefore, the mechanisms underlying the relationship between PhA or IR and age during puberty are thought to extend beyond muscle tissue and BCM and require further detailed investigations.

This study had several limitations. First, as this was a cross-sectional study, whether PhA and IR change with grip strength and advancing age remains unclear. Therefore, future longitudinal studies are required. Second, a recent study indicated that multi-frequency BIA can be a reliable and accurate method for assessing FM and FFM in the real world (i.e., relatively uncontrolled biological factors) [31]. However, although menstruation is thought to have little or no effect on BIA measurement in females [32,33]. Future studies should consider whether it affects PhA and IR. Thirdly, this study compared each variable annually among 15–18-year-olds. However, Japanese boys gained body mass at six-monthly intervals from the age of 15 to 17 years, gaining +1.6 kg, +1.3 kg, +0.9 kg and +0.7 kg respectively (while girls gained between +0.2 kg and +0.6 kg) [34]. Therefore, studies of this period of rapid physical growth, particularly in boys, require analysis at shorter timescales. Finally, while this study measured PhA and IR as indicators of muscle cellular state, it did not observe cellular state directly. As a recent report has indicated that the term ‘muscle quality’ is being used imprecisely [35], future research should focus on both BIA indicators and direct measurements of muscle cellular state.

## Conclusions

This study investigated the relationships among age, PhA, IR, and grip strength in Japanese individuals aged 15–18 years. Significant effects of age were observed for body size, FM, FFM, and grip strength as well as PhA and IR in boys, with significantly higher values observed at age 17 vs. 16 years. Conversely, no significant main effect of age was observed for any of these parameters in the girls. Therefore, while it may be acceptable to present combined values for late adolescent girls, it would be preferable to provide age-specific reference or percentile values for PhA and IR in boys. Furthermore, PhA and IR were significantly correlated with grip strength at each age regardless of sex. This suggests that the PhA and IR can indicate muscle strength in these age groups.

## Supplementary Information



## Figures and Tables

**Table 1:** Mean values and standard deviations for body size, body composition and grip strength by age group.

	Age group (years)				ANOVA	Multiple comparison, Effect size		
		
	15	16	17	18		15 vs. 16 yrs	16 vs. 17 yrs	17 vs. 18 yrs
***Boys***	(n=141)	(n=148)	(n=121)	(n=124)				
*Height (cm)*	168.3±6.3	168.9±5.8	170.4±5.6	169.9±5.5	***F*** **=3.42, ** ***P*** **=0.02**	*P*=1.00, *d*=0.11	*P*=0.25, *d*=0.25	*P*=1.00, *d*=0.08
*Weight (kg)*	56.5±8.4	57.1±7.8	60.2±8.8	59.8±7.6	***F*** **=6.96, ** ***P*** **<0.01**	*P*=1.00, *d*=0.08	***P*** **=0.02, ** ***d*** **=0.37**	*P*=1.00, *d*=0.05
*Body Mass Index (kg/m* *^2^* *)*	19.9±2.5	20.0±2.3	20.7±2.6	20.7±2.3	***F*** **=4.37, ** ***P*** **<0.01**	*P*=1.00, *d*=0.03	*P*=0.10, *d*=0.30	*P*=1.00, *d*=0.00
*Body Fat Mass (kg)*	8.8±4.5	8.0±3.9	9.2±4.4	9.6±3.9	***F*** **=3.52, ** ***P*** **=0.02**	*P*=0.71, *d*=0.18	*P*=0.13, *d*=0.28	*P*=1.00, *d*=0.09
*Fat-free Mass (kg)*	47.7±5.1	49.1±5.6	51.0±5.6	50.2±5.2	***F*** **=9.45, ** ***P*** **<0.01**	*P*=0.16, *d*=0.26	***P*** **=0.04, ** ***d*** **=0.34**	*P*=1.00, *d*=0.14
*Grip Strength (kgf)* *	37.0±7.2	39.1±7.1	41.9±6.7	41.8±7.1	***F*** **=13.56, ** ***P*** **<0.01**	*P*=0.13, *d*=0.27	***P*** **=0.02, ** ***d*** **=0.40**	*P*=1.00, *d*=0.01

***Girls***	(n=75)	(n=76)	(n=61)	(n=92)				

*Height (cm)*	156.6±5.5	157.6±6.2	158.0±5.5	158.5±5.3	*F*=1.55 *P*=0.20	*d*=0.17	*d*=0.07	*d*=0.08
*Weight (kg)*	50.4±6.9	51.2±6.3	50.0±5.9	52.3±6.2	*F*=2.00, *P*=0.11	*d*=0.12	*d*=0.19	*d*=0.38
*Body Mass Index (kg/m* *^2^* *)*	20.5±2.3	20.6±2.1	20.0±1.9	20.8±2.2	*F*=1.80, *P*=0.15	*d*=0.03	*d*=0.29	*d*=0.39
*Body Fat Mass (kg)*	14.2±3.3	14.3±3.8	13.7±3.3	15.2±3.5	*F*=1.91, *P*=0.13	*d*=0.03	*d*=0.16	*d*=0.34
*Fat-free Mass (kg)*	36.3±4.7	36.9±3.9	36.3±3.6	37.1±3.9	*F*=1.16, *P*=0.32	*d*=0.18	*d*=0.17	*d*=0.24
*Grip Strength (kgf)* *	26.3±4.7	26.6±5.3	27.5±4.5	28.2±6.0	*F*=1.73, P=0.16	*d*=0.07	*d*=0.18	*d*=0.13

* Participants for grip strength (boys/girls): 122/61 15-year-old, 126/65 16-year-old, 115/54 17-year-old, and 124/61 18-year-old, respectively.

Values are shown as mean ± standard deviation.

**Table 2:** Mean values and standard deviations for phase angle and impedance ratio in whole-body, upper limbs and lower limbs by age group.

	Age group (years)				ANOVA	Multiple comparison & Effect size		
		
	15	16	17	18		15 vs. 16 yrs	16 vs. 17 yrs	17 vs. 18 yrs
***Boys***	(n=141)	(n=148)	(n=121)	(n=124)				
***Phase Angle (°)***								
* * *Whole body*	5.93±0.54	5.99±0.57	6.25±0.57	6.20±0.58	***F*** **=9.71, ** ***P*** **<0.01**	*P*=1.00, *d*=0.11	***P*** **<0.01, ** ***d*** **=0.45**	*P*=1.00, *d*=0.09
* * *Upper limb*	6.01±0.51	6.09±0.56	6.41±0.58	6.28±0.54	***F*** **=14.03, ** ***P*** **<0.01**	*P*=1.00, *d*=0.15	***P*** **<0.01, ** ***d*** **=0.55**	*P*=0.44, *d*=0.23
* * *Lower limb*	6.20±0.66	6.28±0.61	6.49±0.63	6.53±0.73	***F*** **=8.00, ** ***P*** **<0.01**	*P*=1.00, *d*=0.14	***P*** **=0.04, ** ***d*** **=0.34**	*P*=1.00, *d*=0.06

***Impedance Ratio***								

* * *Whole body*	0.791±0.018	0.789±0.019	0.780±0.020	0.780±0.020	***F*** **=11.07, ** ***P*** **<0.01**	*P*=1.00, *d*=0.11	***P*** **<0.01, ** ***d*** **=0.44**	*P*=1.00, *d*=0.00
* * *Upper limb*	0.796±0.017	0.794±0.018	0.783±0.019	0.787±0.018	***F*** **=17.57, ** ***P*** **<0.01**	*P*=1.00, *d*=0.13	***P*** **<0.01, ** ***d*** **=0.65**	*P*=0.55, *d*=0.22
* * *Lower limb*	0.782±0.022	0.779±0.021	0.772±0.021	0.770±0.024	***F*** **=8.88, ** ***P*** **<0.01**	*P*=1.00, *d*=0.14	***P*** **=0.03, ** ***d*** **=0.34**	*P*=1.00, *d*=0.07

***Girls***	(n=75)	(n=76)	(n=61)	(n=92)				

***Phase Angle (°)***								
* * *Whole body*	5.21±0.55	5.25±0.55	5.14±0.53	5.23±0.54	*F*=0.53, *P*=0.67	*d*=0.06	*d*=0.21	*d*=0.17
* * *Upper limb*	5.35±0.58	5.41±0.54	5.39±0.52	5.34±0.56	*F*=0.232, *P*=0.87	*d*=0.11	*d*=0.03	*d*=0.09
* * *Lower limb*	5.55±0.56	5.60±0.64	5.38±0.57	5.59±0.70	*F*=1.70, *P*=0.17	*d*=0.08	*d*=0.36	*d*=0.32

***Impedance Ratio***								
* * *Whole body*	0.817±0.019	0.815±0.020	0.820±0.019	0.816±0.018	*F*=0.90, *P*=0.44	*d*=0.11	*d*=0.25	*d*=0.24
* * *Upper limb*	0.819±0.019	0.817±0.018	0.818±0.017	0.815±0.018	*F*=0.69, *P*=0.56	*d*=0.14	*d*=0.10	*d*=0.16
* * *Lower limb*	0.805±0.019	0.804±0.022	0.811±0.019	0.804±0.024	*F*=1.99, *P*=0.12	*d*=0.07	*d*=0.37	*d*=0.35

**Table 3:** Correlations between whole-body PhA or IR and other variables.

	Boys		Girls	

	Phase angle	Impedance ratio	Phase angle	Impedance ratio
*Height*	0.05	−0.08	−0.09	0.07
*Weight*	0.46**	−0.48**	0.27**	−0.27**
*BMI*	0.51**	−0.52**	0.37**	−0.36**
*Body fat mass*	0.15**	−0.18**	0.04	−0.05
*Fat-free mass*	0.58**	−0.58**	0.44**	−0.43**
*Grip strength*	0.54**	−0.54**	0.51**	−0.50**

(Values are correlation coefficient (r).

** *P*<0.01).
