# Supplementary material for: Relationship Between Age and Grip Strength, Phase Angle, and Impedance Rate Measured by Bioelectrical Impedance Analysis in Japanese Individuals Aged 15–18 Years
Source: Physiol Res. 2026 Jun 1;75(3):545–55. doi: 10.33549/physiolres.935774 (PMC13557532; doi:10.33549/physiolres.935774)
Supplement: Supplementary file 1 [file 75_545_Suppl_Table_1.docx]

**Supplementary Table 1.** Percentile values for phase angle and impedance rate in boys (by age) and girls.

|  |  | **Boys** | | | | | **Girls** |
| --- | --- | --- | --- | --- | --- | --- | --- |
|  |  | **15 years** | **16 years** | **17 years** | **18 years** | **All** | **All** |
| ***Phase Angle (°)*** |  |  |  |  |  |  |  |
| *Whole body* | 95^th^ | 6.77 | 6.97 | 7.20 | 7.14 | 7.06 | 6.15 |
|  | 75^th^ | 6.34 | 6.40 | 6.62 | 6.56 | 6.48 | 5.59 |
|  | 50^th^ | 5.88 | 6.01 | 6.21 | 6.19 | 6.05 | 5.21 |
|  | 25^th^ | 5.56 | 5.54 | 5.86 | 5.76 | 5.67 | 4.79 |
|  | 5^th^ | 5.11 | 5.12 | 5.40 | 5.38 | 5.20 | 4.36 |
|  | 95%CI | 5.84-6.02 | 5.90-6.09 | 6.15-6.35 | 6.09-6.30 | 6.03-6.13 | 5.15-5.27 |
| *Upper limb* | 95^th^ | 6.93 | 6.99 | 7.36 | 7.15 | 7.15 | 6.32 |
|  | 75^th^ | 6.38 | 6.46 | 6.87 | 6.65 | 6.56 | 5.75 |
|  | 50^th^ | 5.95 | 6.08 | 6.37 | 6.26 | 6.15 | 5.35 |
|  | 25^th^ | 5.70 | 5.66 | 5.97 | 5.89 | 5.78 | 4.97 |
|  | 5^th^ | 5.28 | 5.20 | 5.60 | 5.48 | 5.30 | 4.56 |
|  | 95%CI | 5.92-6.09 | 6.00-6.18 | 6.60-6.51 | 6.18-6.37 | 6.14-6.23 | 5.31-5.43 |
| *Lower limb* | 95^th^ | 7.44 | 7.24 | 7.48 | 7.62 | 7.44 | 6.58 |
|  | 75^th^ | 6.70 | 6.76 | 6.96 | 7.02 | 6.84 | 5.90 |
|  | 50^th^ | 6.16 | 6.31 | 6.47 | 6.54 | 6.36 | 5.54 |
|  | 25^th^ | 5.75 | 5.84 | 6.02 | 6.03 | 5.91 | 5.09 |

CI; Confidence Interval.
